# Rhythmic Haptic Cueing Using Wearable Devices as Physiotherapy for Huntington Disease: Case Study

**DOI:** 10.2196/18589

**Published:** 2020-09-14

**Authors:** Theodoros Georgiou, Riasat Islam, Simon Holland, Janet van der Linden, Blaine Price, Paul Mulholland, Allan Perry

**Affiliations:** 1 School of Mathematical and Computer Sciences Heriot-Watt University Edinburgh United Kingdom; 2 School of Computing and Communications The Open University Milton Keynes United Kingdom; 3 Knowledge Media Institute The Open University Milton Keynes United Kingdom; 4 PJ Care Limited Peterborough United Kingdom

**Keywords:** physiotherapy, rhythm, haptic, tactile, wearable, cueing, Huntington, gait

## Abstract

**Background:**

Huntington disease (HD) is an inherited genetic disorder that results in the death of brain cells. HD symptoms generally start with subtle changes in mood and mental abilities; they then degenerate progressively, ensuing a general lack of coordination and an unsteady gait, ultimately resulting in death. There is currently no cure for HD. Walking cued by an external, usually auditory, rhythm has been shown to steady gait and help with movement coordination in other neurological conditions. More recently, work with other neurological conditions has demonstrated that haptic (ie, tactile) rhythmic cues, as opposed to audio cues, offer similar improvements when walking. An added benefit is that less intrusive, more private cues are delivered by a wearable device that leaves the ears free for conversation, situation awareness, and safety. This paper presents a case study where rhythmic haptic cueing (RHC) was applied to one person with HD. The case study has two elements: the gait data we collected from our wearable devices and the comments we received from a group of highly trained expert physiotherapists and specialists in HD.

**Objective:**

The objective of this case study was to investigate whether RHC can be applied to improve gait coordination and limb control in people living with HD. While not offering a cure, therapeutic outcomes may delay the onset or severity of symptoms, with the potential to improve and prolong quality of life.

**Methods:**

The approach adopted for this study includes two elements, one quantitative and one qualitative. The first is a repeated-measures design with three conditions: before haptic rhythm (ie, baseline), with haptic rhythm, and after exposure to haptic rhythm. The second element is an in-depth interview with physiotherapists observing the session.

**Results:**

In comparison to the baseline, the physiotherapists noted a number of improvements to the participant’s kinematics during her walk with the haptic cues. These improvements continued in the after-cue condition, indicating some lasting effects. The quantitative data obtained support the physiotherapists’ observations.

**Conclusions:**

The findings from this small case study, with a single participant, suggest that a haptic metronomic rhythm may have immediate, potentially therapeutic benefits for the walking kinematics of people living with HD and warrants further investigation.

## Introduction

### Background

Rhythmic cueing is a technique that is able to provide immediate improvements to asymmetrical or irregular gait for a variety of neurological conditions [1-5]. This paper presents a case study of the first reported application, to the best of our knowledge, of rhythmic cueing via haptics (ie, through the sense of touch) for a participant diagnosed with Huntington disease (HD).

HD is an inherited genetic disorder that results in the death of brain cells [6]. HD has a relatively low occurrence: around 10.6-13.7 individuals per 100,000 in western populations, and 1-7 individuals per million in Asian populations [7]. Even so, George Huntington, the person who first defined the disease in 1872, described it as follows: “Once it begins, it clings to the bitter end” [8]. There is currently no cure for HD [6].

Specific symptoms of HD vary between people [9]; however, HD symptoms generally start with subtle changes in mood and mental abilities [9]. These symptoms are then followed by a general lack of coordination and an unsteady gait [10]. In later stages of the disease, uncoordinated, jerky body movements become more apparent [9]. *Huntington* *chorea*—chorea, or χορεία, being the ancient Greek name for *dance*—is a name given to the hand and feet movements caused by HD because of their unfortunate loose resemblance to dancing.

Physical abilities gradually worsen, until coordinated movement becomes difficult, eventually affecting the person’s vocal cords, making them unable to talk [9,10]. Eventually, due to the gradual death of brain cells, mental abilities often decline into dementia [11]. Since HD is a genetic disease, symptoms can start at any age. However, symptoms do not usually become apparent until a person is between 30 and 50 years of age [6,11].

A small percentage of HD cases (about 8%) start before the age of 20 years, and typically present with symptoms more similar to Parkinson disease [11]. This is often defined as juvenile HD (JHD), and it differs in that it generally progresses faster, with affected individuals usually remaining alive no longer than around 10 to 15 years after signs and symptoms appear [12]. In such cases, chorea is generally exhibited only briefly, if at all. Additional signs of JHD include slow movements, clumsiness, frequent falling, rigidity, slurred speech, and drooling. A total of 30%-50% of persons with JHD often experience seizures [12,13].

Even though there is currently no cure [6], treatments [11] and frequent physiotherapy [14] can relieve some symptoms and, in some cases, improve quality of life [11]. Generally, full-time care is required in the later stages of the disease [10].

In the case of neurological conditions that affect gait more generally, asking survivors to match their steps to a steady external rhythm has been found to improve various gait characteristics, as we will now outline. This method of gait-related physiotherapy and rehabilitation, primarily using audio rhythms, has been widely explored in conditions such as hemiparetic stroke [15], cerebral palsy [16], and Parkinson disease [17,18] with promising results. More recently, steady rhythms mediated through the haptic, as opposed to auditory, modality (ie, mediated via the sense of touch) have been demonstrated to show very similar therapeutic benefits [5]. In some contexts, rhythmic haptic cueing (RHC) can have advantages over audio cueing, as it can be less obtrusive and leaves the sense of hearing free for other purposes [19].

In this case study, we tested RHC for assisting and enhancing current physiotherapy practices of HD and JHD. Even though physiotherapy for HD does not aim to fully restore walking abilities, it can have long-term therapeutic effects in delaying the disease’s progression; this could extend the period that a person with HD can be independently mobile, hence, providing better quality of life.

Results from a single participant with JHD in this study were analyzed qualitatively by an independent team of expert physiotherapists. They commented on the participant’s gait pattern and characteristics before cueing, during application of the rhythmic cue, and immediately after cueing.

### Human Gait

Before moving on to the details of the study and the results obtained, it may be useful to clarify aspects of human gait and kinematics, including terminology used by physiotherapists to describe gait [20].

**Figure 1:**
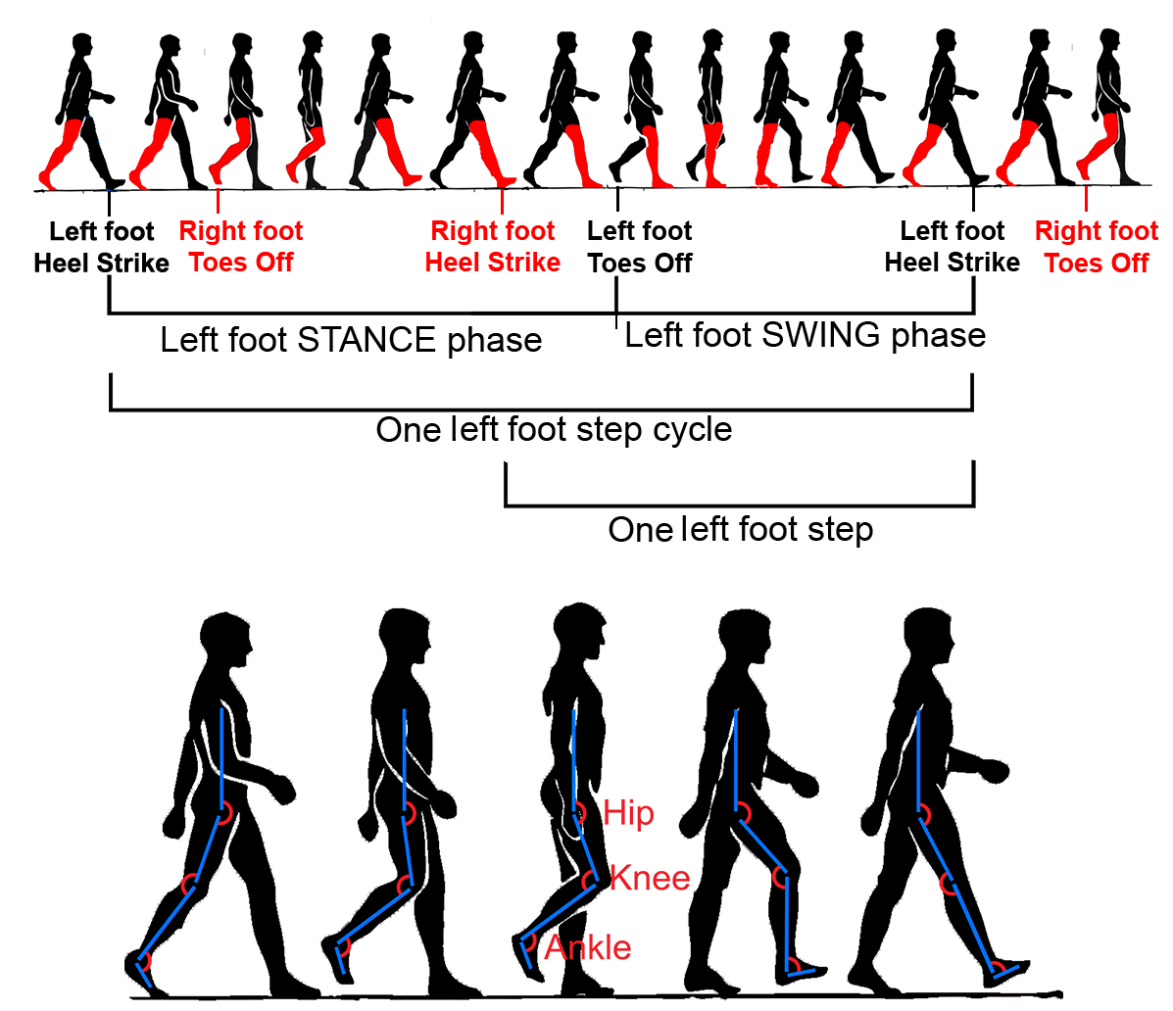
Illustration of the human gait. The phases in human gait are shown at the top, and the joint flexions during one step are shown at the bottom.

Each step, or step cycle, consists primarily of two phases: the swing phase and the stance phase. The swing phase, as the name suggests, happens from the moment the toes of the foot initiating a given step lift off the ground and the leg begins to swing forward (see Figure 1). This phase completes when the heel of that foot strikes the ground, beginning to support the body’s weight, thereby starting the stance phase (see Figure 1). Between each of two successive step cycles of alternating legs, there is what is known as the double-support phase, where both legs touch the ground, as illustrated in Figure 1.

The three principal lower-limb joints flex and extend during each step; these are the hip, the knee, and the ankle joint. A joint is said to flex during reduction of the joint angle and to extend when the angle increases. The relevant flexions and extensions of these joints during a typical step are shown at the bottom of Figure 1.

All of the above aspects of gait can be affected by neurological conditions such as HD, which affect the motor control centers in the brain. Precise effects can vary greatly depending both on the condition and the individual case. However, all of these conditions generally affect the activation patterns of one or more muscle groups, reducing the flexion capabilities of the lower-limb joints.

As briefly discussed in the sections below, walking to a steady rhythm can have various gait-related benefits. The next section considers entrainment, the underlying neurological mechanism that makes walking to a rhythm possible.

### Entrainment

In physics, entrainment is a natural phenomenon where two or more periodic processes interact with each other to adjust to a common or related period. In the early 1990s, biological and specifically human aspects of entrainment were investigated in some detail. Studies showed that gait can be facilitated using rhythmic stimulation [1,21,22]. With these early studies, human capacity for biological entrainment became better understood, and applications for movement rehabilitation of neurological conditions were studied in more detail.

Applications included the use of auditory cues to synchronize human motor coordination into more stable temporal patterns. In such cases, entrainment mechanisms act between the external rhythm and the motor response to stabilize and regulate gait patterns [15].

### Rhythmic Haptic Cueing

Haptics is a term used when referring to any form of communication involving the sense of touch [23] and can provide a valuable mode for mediating rhythmic cueing for entrainment and motor movement physiotherapy. RHC shows great potential in physiotherapy and rehabilitation, with similar and immediate benefits to the more established auditory cue; however, benefits from RHC can be achieved less obtrusively, while leaving the ears free for improved safety, social integration, and situation awareness [5,24]. Generally in computing, particularly with mobile and wearable devices, uses of haptics are typically limited to notifications, such as alerts about incoming phone or text messages. This common mode of usage engages with relatively simple human stimulus-response mechanisms. By contrast, the mode of the considered use primarily does not engage with cognitively mediated nor reflex versions of stimulus-response mechanisms, but does engage with different human mechanisms that mediate human entrainment [25] and that are predictive rather than simply reactive [26]. Due to physiological delays, stimulus response is not a viable way to synchronize to rhythm [26].

The human capacity to entrain, on the other hand, can provide the fine-grained synchronization that allows movement to be coordinated, both physically and mentally [5], in synchrony to an external rhythm.

**Figure 2:**
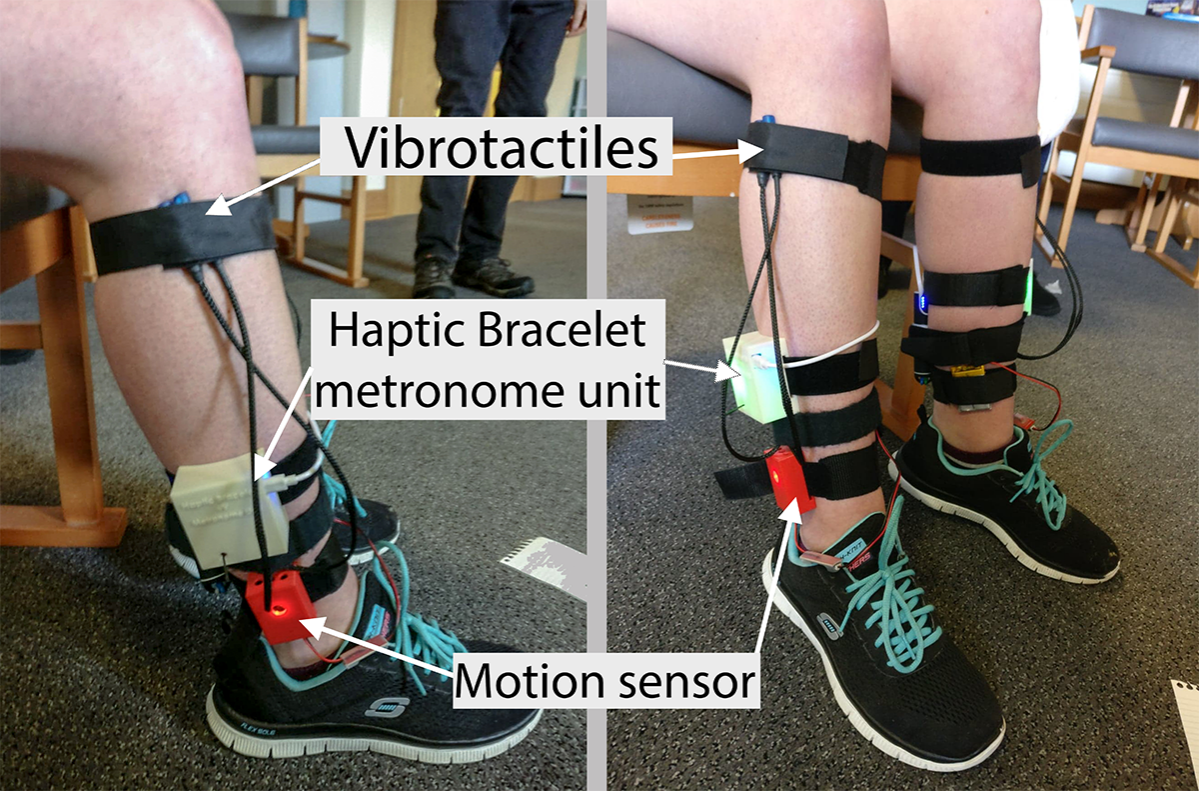
The Haptic Bracelet wearable devices placed on the participant’s legs (one device on each leg). The vibrotactiles used for delivering the haptic cues were strapped on near the knee using Velcro straps.

In this study, RHC was presented to the participant via the Haptic Bracelet system [5]. The Haptic Bracelet system is made up of prototype lightweight wearable devices, capable of providing RHC in the form of vibrations through carefully calibrated vibrotactiles on alternating legs, leaving the audio channel clear and free of distractions (see Figure 2). The Haptic Bracelet system includes a vibrotactile unit that produces the RHC. A vibrotactile unit is worn on each leg using Velcro straps. Each vibrotactile unit consists of an Arduino microprocessor board, a Wi-Fi board to communicate with an external control unit, a vibrotactile actuator, and vibrotactile motor drivers. The control unit consists of bespoke computer software that runs on a laptop and communicates with the vibrotactile unit via a Wi-Fi network. The researchers operate the system using the control unit. The Haptic Bracelet system also includes a motion sensor employing inertial measurement units that can track the motion of the participant as she walks on the runway. Detailed technical specifications of the Haptic Bracelet system have been previously published [5].

The next sections describe the methodology used and the initial results obtained.

## Methods

### Study Design

The approach adopted for this study was a repeated-measures design that included *before* (ie, baseline), *with*, and *after* conditions, followed by an in-depth interview with a group of physiotherapists observing the session.

### Research Participant and Setting

For this case study, in order to investigate the effects of RHC in the gait of people with HD, one female participant (RK; this is a pseudonym for the participant) was recruited from the PJ Care residential care home in the United Kingdom. The recruitment criteria included being diagnosed with HD, over 18 years old, and able to walk 10 meters independently. RK was 28 years old when the study was performed, and she was diagnosed with JHD at a young age. RK is a tip toe walker—physiotherapy partially addressed this. However, frequent falls she recently experienced affected her confidence, causing her to be wheelchair bound when traveling outdoors. Her carer was present during the study for safeguarding.

RK provided written informed consent prior to this study. This study was conducted indoors within PJ Care’s residential care center. The room used was large enough to accommodate a 10-meter-long, straight-line walking runway while at the same time allowing a group of physiotherapists to observe the walks. This room had carpeting that covered the entire floor.

The study received ethical approval from the London-Stanmore Research Ethics Committee of the National Health Service – Health Research Authority (17/LO/2050) and from The Open University’s Human Research Ethics Committee (HREC/2017/2633/Holland1).

### Procedure

#### Prebaseline

The participant (RK) was first asked to walk the length of a 10-meter runway six times without wearing the bracelets as a *prebaseline* measure. She was asked to walk as she normally would, allowing for the group of physiotherapists present to view how she walked without wearing any devices. These *prebaseline* walks made it possible to investigate whether wearing the devices would have any impact on her walk, even without cueing.

#### Baseline

For the next stage (ie, baseline), the Haptic Bracelet system components were attached to each leg, but without providing haptic cueing. RK was then asked to walk the length of a 10-meter runway six times (three times each way) to provide a baseline measure. The motion sensor unit tracked the motion for baseline measurements. A carer walked alongside RK for safety. A chair was placed on either end of the runway for RK to rest if needed between trials. Short breaks were scheduled between each session, but we also made clear to RK and her carer that they could request a break at any point during the study, even midtrial.

#### Familiarization Period

After completing the baseline set of trials without haptic cueing, the tactile metronome of the Haptic Bracelet system was switched on with RK simply sitting on a chair to feel the tactile cues. For the purpose of initial familiarization, a slow but arbitrary cueing rate was set. The tactile cue intensity was adjusted so that pulses could be felt clearly but without causing any discomfort. The placement of the vibrotactiles delivering the cues had been previously decided after consulting with physiotherapists during earlier studies [5,27] with stroke and brain injury survivors (see Figure 2 for vibrotactile placement).

Once the intensity was set to a comfortable level, the period of the metronome cue was adjusted for the participant to match her natural walking speed, as observed and calculated from the baseline condition. Setting the metronome’s period to match the individual’s natural walking rhythm is considered important for rhythm-based gait rehabilitation, generally, and for other conditions, as this approach has been found to help participants feel most comfortable in timing their steps to the beat of the rhythm [28].

Once the tactile intensity and the metronome period was adjusted, RK was asked to stand up and try to step in place following the metronome’s rhythm without moving forward. At this stage, RK was asked again if she felt like she needed any further adjustments to be made on the metronome period or the vibrotactile intensity; we adjusted accordingly.

#### With-Cue and After-Cue Conditions

As previously noted, in addition to serving as a basis for comparison, the baseline measurements were used to establish a reference cadence for setting the tempo of the haptic cues for the *with-cue* condition. The with-cue condition consisted of six walks with haptic cueing switched on.

After a short 5-minute break, RK was invited to walk six more times while trying to walk to the rhythm from memory (ie, the *after-cue* condition). At this stage, RK has been walking for around 15 minutes in total, excluding break time. Even though RK was eager to walk, she exhibited higher levels of fatigue than we had anticipated, and her carer asked if we could end the session after four walks in the after-cue condition. In the interest of our patient’s safety, we immediately complied.

#### Interview With Physiotherapy Experts

A group of five experienced and specialized physiotherapists were present in the room to observe the participant during all four conditions and to make detailed notes on their observations. After RK left the room, we held an in-depth interview with this group at the end of the session to discuss their observations. The physiotherapists also provided a formal gait-assessment report for each stage of this study, as shown in Table 1.

**Table 1:** Gait-assessment report by physiotherapists.

Phase	Participant (RK) movement	Observations
Prebaseline	RK mobilized 10 meters six times without wearing the haptic device.	Reduced hip flexion and no heel strike on stance phase Reduced hip and knee flexion during midswing Reduced toe-off Difficulty with turnings
Baseline	RK mobilized 10 meters six times while wearing the device that was not switched on.	Reduced hip flexion and no heel strike on stance phase Reduced hip and knee flexion during midswing Reduced toe-off Difficulty with turnings
With cues	RK mobilized 10 meters six times while wearing the device that was switched on.	Increased hip flexion and has a slight heel strike on stance Increased knee flexion during midswing Increased hip flexion, knee flexion, and toe-off, which help her clear the ground
After cues	RK mobilized 10 meters four times while wearing the device that was switched off to observe whether she was able to remember the rhythm.	Retains changes from previous trial Cannot fully comment on this, as this requires several trials to ascertain her ability to remember the sensation from the device by observing it through her gait pattern

## Results

### Observations From the Physiotherapists

Observations focused primarily on how RK’s joint angles—hip, knee, and ankle (see Figure 1)—changed during her walking between conditions. The physiotherapists provided a gait-assessment report regarding RK for each phase of the study as summarized below in Table 1.

The gait patterns were further discussed in the interview following the walking trials. The two most senior physiotherapists in the team (ZN and AF; these are pseudonyms for these two PJ Care physiotherapists) led the conversation describing how RK walked in the prebaseline session (ie, walk with no devices). Specifically, both ZN and AF agreed that RK walked with reduced hip flexion causing her to land on the front part of her foot first (ie, toes area) showing no, or limited, heel strike during the beginning of her stance phase. ZN said, “RK has reduced hip flexion; knee flexion has reduced as well. There is decreased dorsi flexion, that’s why she doesn’t have any heel strike,” and added, “reduced toe-off and not clearing the ground properly.” According to the physiotherapists, RK showed reduced hip flexion during the swing phase and reduced knee flexion midswing, where knee flexion is normally at maximum. This caused RK to experience difficulties clearing the ground with her toes and may be a factor contributing to the frequent falls she is experiencing.

ZN and AF commented that there was no difference in the way that RK walked between the baseline and prebaseline conditions. This indicates that wearing the devices when switched off did not affect the way RK walked. However, both ZN and AF agreed that when the haptic metronome was switched on, and the devices gave RK a tactile cue on alternating legs matching her preferred pace, RK’s hip flexion increased, giving her a slight heel strike at the beginning of her stance phase. ZN said the following:

I think when you put the bracelet [switch on haptic metronome] she has improved, because you can really see that there is a bit of hip flexion in there and then knee flexion. But there is no dorsi flexion; maybe it’s just the point of her condition that’s deteriorating. However, I saw that she also clears the ground more and because in Huntington’s the basal ganglia [part of the brain] is affected and so gait initiation is difficult. That’s why RK was swaying from side to side before making her first step.

ZN then added, “The rhythm is helping with the initiation,” and concluded, “The rhythm has helped with the flexion values; it is minimal but compared to baseline has improved.”

Another change observed in this condition was that RK’s knee flexion increased midswing, while her overall hip and knee flexion increased, allowing RK to have a better toe-off, clearing the ground better.

During the after-cue condition, when RK walked to the rhythm from memory (ie, when the haptic metronome was switched off again), the physiotherapists commented on how her walk pattern remained the same as in the with-cue condition. ZN said, “It’s the same as I observed [as with-cue condition]. She has increased hip flexion, improved knee flexion but not dorsi flexion; she clears the ground better.” RK’s retaining of rhythm from memory is consistent with studies relating to other conditions [5] and suggests evidence of rhythm persistence, where a participant retains the rhythm in her head, and sustains gait improvements for a short period after cueing. This phenomenon has been observed in haptic cueing for the purpose of improving gait with other neurological conditions, particularly hemiparesis [5].

In addition to commenting on the changes in RK’s gait, the physiotherapists explained how flexion and changes in joint angles are more relevant to HD rather than gait symmetry, which was important for hemiparetic stroke survivors as investigated in Georgiou et al [29]. They also mentioned that it seemed to them that “the devices have improved balance.” For people living with HD, risk of falls is very high, and improving balance can help in reducing the frequency of falls. The physiotherapists observed that both when walking with the haptic metronome turned on and immediately afterward without the metronome, it seemed that RK had a better sense of balance when walking.

The physiotherapists also commented about the key parameters that can be useful to measure when using the wearable sensors to support the visual assessment. They have suggested measuring speed of walking, stride length, and joint angle kinematics for flexion angles to better understand toe-off and heel strike events. They also suggested, if it was possible, to measure “how far someone has walked,” adding “possibly outdoor walking.”

They provided critical feedback on certain issues that this study could not address; for example, the issue of turning and fatigue. They observed that RK was more comfortable with straight-line walking than turning. The nature of RHC is such that, at this stage, it can facilitate straight-line walking but cannot address turning. Also, the physiotherapists observed that RK was getting fatigued during the study, and RK’s carer suggested to end the study before she could complete the six trials for the after-cue condition. However, it was not possible to determine the actual reason for RK’s fatigue. It was not clear whether, due to RHC, the cost of energy of walking was increased, whether RK got fatigued as she was not used to walking independently, or whether she felt pressure to perform better while being observed.

### Data From the Motion Sensors

The motion sensors used in this study consisted of two inertial measurement units, one placed on each ankle of the participant. The data were sent to the control unit software running on the laptop via the Wi-Fi network. The data obtained were analyzed using bespoke algorithms running on MATLAB (The MathWorks, Inc) [5].

**Figure 3:**
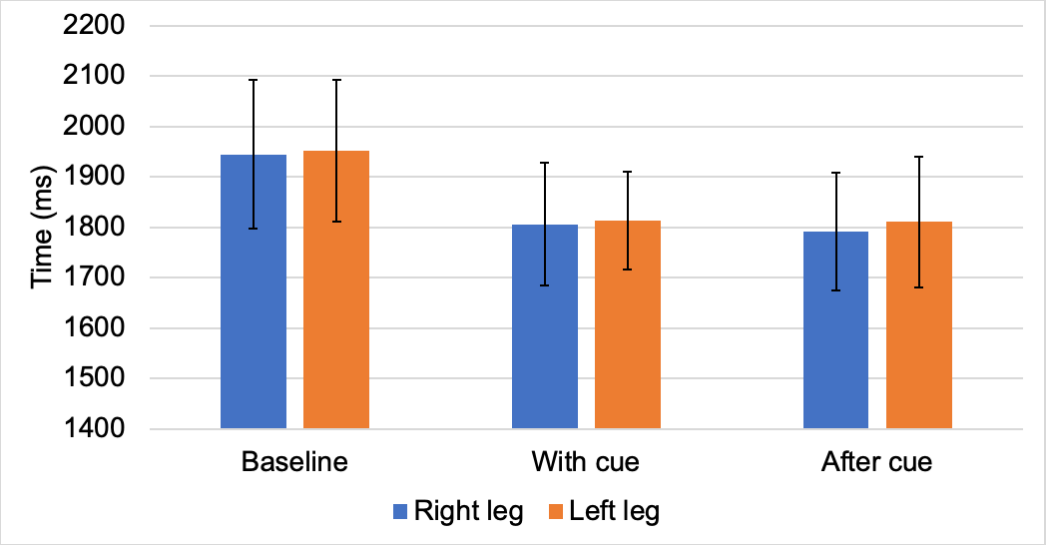
Temporal gait parameters (stride cycle time) for both legs in the baseline, with-cue, and after-cue conditions.

The following results show the temporal gait parameters for both legs in the baseline, with-cue, and after-cue conditions. Figure 3 shows the stride cycle time for both legs for the three experimental conditions. It is clear from the results that due to RHC, the time taken to complete a stride was reduced for both legs; in addition, the same rate was retained for the after-cue condition. This further supports the observations of the physiotherapists that RHC has changed RK’s gait pattern and she has been able to retain the changes from memory.

The motion sensors used in this study could not measure the change in flexion and joint angles on which the physiotherapists commented. To measure such changes on joint angle kinematics, a different motion-tracking facility is required, such as a 3D, optical, motion-capture system or a seven-sensor inertial measurement unit system [30].

## Discussion

### Principal Findings

In this preliminary study, a single participant with JHD walked indoors following a steady rhythm. The steady rhythm was delivered haptically through carefully controlled tactile cues on alternating legs at a cadence set to match the participant’s preferred pace, as measured during initial baseline trials. The participant’s gait was visually observed by a group of experienced physiotherapists.

The team of physiotherapists reported changes in the flexion of RK’s joints, rather than changes such as improved spatial and temporal symmetry, which are typically reported for other neurological conditions such as hemiparesis [5,31].

In this study, motion sensors were used to measure temporal gait parameters for the walking trials. Results from temporal data show both that RHC was associated with changes in gait pattern when the Haptic Bracelet system was switched on and that RK could retain a similar improved walking pattern after the cue was withdrawn. One of the limitations of this study is that the two ankle-worn motion sensors were not designed to measure all of the parameters relevant to all of the changes in gait kinematics that the physiotherapists’ observed. A more sophisticated motion-tracking system would be required to measure changes in gait kinematics, such as joint angles. However, this was not practical in the case of this study. A potential alternative could be to use a portable, wearable, motion-tracking setup using seven inertial measurement units [30].

The team of physiotherapists concluded that RK assumed generally better walking kinematics, exhibiting better joint flexion, during both the with-cue and the after-cue conditions. This allowed for better ground clearance, potentially reducing RK’s risk of falling.

Even though RHC can potentially improve gait features for straight-line walking, RHC's effect on turning is not clearly understood. The physiotherapists observed that RK was facing difficulties while turning. It is not yet clear whether the RHC was interfering with RK’s turning motion or whether RK was being extra cautious, as people tend to have a higher risk of falls while turning [32]. RK might lack confidence in turning due to her history of frequent falls.

The physiotherapists observed that RK was experiencing fatigue toward the end of the study. It is not well understood whether RHC can be a contributing factor to fatigue. Other factors may cause fatigue; HD itself, for example, can be a contributing factor to fatigue, as this is one of the symptoms of the disease. Other possible causes of fatigue could be RK’s overall lack of physical activity leading to low stamina or RK putting more effort into her walks because she was being observed. However, in general terms, it is not clearly understood whether RHC can increase the cost of energy of walking.

### Conclusions

The observations from this preliminary study suggest that RHC may have immediate benefits for walking among individuals with HD, potentially extending the period of independent mobility for people with HD. This warrants further investigation.

### Further Work

One of the limitations of the study is that there was only a single participant. Previous research on RHC using wearable devices has shown immediate changes to gait pattern for people living with neurological conditions such as hemiparesis [29]. To the best of our knowledge, this study is the first step in investigating how RHC using wearable devices can help people living with HD. A number of lessons can be drawn from this preliminary work that can feed forward to future research in this field.

Further studies with more participants with HD or JHD can be conducted to investigate whether the immediate benefits observed in this study can be replicated in others. Long-term studies can also be conducted to investigate whether the immediate benefits of RHC can be sustained for a longer period of time and how this might lead to extended periods of independent mobility for people with HD or JHD.

## Appendix

Multimedia Appendix 1 Supplementary data: baseline stride time.

Multimedia Appendix 2 Supplementary data: with-cue stride time.

Multimedia Appendix 3 Supplementary data: after-cue stride time.

## Abbreviations

HD: Huntington disease
JHD: juvenile Huntington disease
RHC: rhythmic haptic cueing
